# GPCRs from *fusarium graminearum* detection, modeling and virtual screening - the search for new routes to control head blight disease

**DOI:** 10.1186/s12859-016-1342-9

**Published:** 2016-12-15

**Authors:** Emmanuel Bresso, Roberto Togawa, Kim Hammond-Kosack, Martin Urban, Bernard Maigret, Natalia Florencio Martins

**Affiliations:** 1 EMBRAPA Genetic Resources and Biotechnology, Brasília, DF 70770-917 Brazil; 2 Department of Plant Biology and Crop Science, Rothamsted Research, Harpenden, Hertfordshire, AL5 2JQ UK; 3 CAPSID Team, LORIA, UMR 7503, CNRS, Lorraine University, Vandœuvre-lès-Nancy, 54506 France

**Keywords:** G-protein coupled receptors, *Fusarium graminearum*, Structural bioinformatics, Fusarium head blight

## Abstract

**Backgound:**

*Fusarium graminearum* (FG) is one of the major cereal infecting pathogens causing high economic losses worldwide and resulting in adverse effects on human and animal health. Therefore, the development of new fungicides against FG is an important issue to reduce cereal infection and economic impact. In the strategy for developing new fungicides, a critical step is the identification of new targets against which innovative chemicals weapons can be designed. As several G-protein coupled receptors (GPCRs) are implicated in signaling pathways critical for the fungi development and survival, such proteins could be valuable efficient targets to reduce *Fusarium* growth and therefore to prevent food contamination.

**Results:**

In this study, GPCRs were predicted in the FG proteome using a manually curated pipeline dedicated to the identification of GPCRs. Based on several successive filters, the most appropriate GPCR candidate target for developing new fungicides was selected. Searching for new compounds blocking this particular target requires the knowledge of its 3D-structure. As no experimental X-Ray structure of the selected protein was available, a 3D model was built by homology modeling. The model quality and stability was checked by 100 ns of molecular dynamics simulations. Two stable conformations representative of the conformational families of the protein were extracted from the 100 ns simulation and were used for an ensemble docking campaign. The model quality and stability was checked by 100 ns of molecular dynamics simulations previously to the virtual screening step. The virtual screening step comprised the exploration of a chemical library with 11,000 compounds that were docked to the GPCR model. Among these compounds, we selected the ten top-ranked nontoxic molecules proposed to be experimentally tested to validate the *in silico* simulation.

**Conclusions:**

This study provides an integrated process merging genomics, structural bioinformatics and drug design for proposing innovative solutions to a world wide threat to grain producers and consumers.

**Electronic supplementary material:**

The online version of this article (doi:10.1186/s12859-016-1342-9) contains supplementary material, which is available to authorized users.

## Background

The ascomycete *Fusarium graminearum* (FG) is a filamentous fungus dwelling on and in a wide range of plant species, on crop debris and within soil. This fungus causes Fusarium head blight (FHB) disease on wheat, barley. FG is also responsible for various corn and rice diseases [1]. FG is a highly destructive pathogen of cereals reduces grain quality rather than grain production. FG causes two main problems: first, seed quality is reduced, and secondly, infection produces mycotoxin-contaminated grains. Among the various sesquiterpenoid trichothecene toxins produced by FG, deoxynivalenol, also known as vomitoxin, is one of the most important [2]. Deoxynivalenol contaminated grains are often considered unfit for animals and/or human consumption leading to considerable economic losses [3, 4].

Fungicide applications are only moderately effective at controlling FHB and often intrinsic resistance problems have been encountered [5, 6]. The identification of new fungicides is urgently required to limit FG development. In the search for new, efficient and selective fungicides able to control the development of the pathogen, the first step is to find relevant targets [7, 8].

G-protein coupled receptors (GPCRs) are the starting point for the control of several signaling pathways and are therefore considered a potentially rich source of innovation as drug targets and for drug design to alleviate many human diseases of genetic and / or biotic origins [9]. GPCRs, which are activated by a large panel of factors ranging from light, small amines to hormones and chemokines, initiate signaling cascades resulting in multiple cell responses. GPCRs constitute a large family of proteins, the signature of which consists of a transmembrane domain embedded within the plasma membrane and possess seven transmembrane helices. Their functions are to detect extracellular signals and to activate intracellular mediated signal transduction pathways and appropriate cellular responses. GPCRs classically transmit a signal via the activation of heterotrimeric G proteins. The sustained stimulation leads to the activation of G protein-coupled receptor kinases and the recruitment of arrestin proteins, which engage alternative signaling pathways [10].

In fungi, GPCRs are known to be implicated in biological processes including vegetative growth, sporulation, stress responses and pathogenicity [11]. GPCRs have been the subject of numerous bioinformatics studies to explore their potential suitability as drug targets [12]. As a result, the entire set of GPCRs encoded by various fungi has been predicted for several fungi including *Saccharomyces cerevisae*, *Schizosaccharomyces pombe*, the saprobes *Aspergillus spp*., *Neurospora crassa*, and *Trichoderma spp*., the plant pathogens *Magnaporthe grisea* and *Verticillium spp*., and the human/animal infecting pathogen *Cryptococcus neoformans* [13–21]. Ma et al. previously explored the GPCRs repertoire for *Fusarium* species, but only sequence alignments were used for these predictions [22]. As GPCRs are known not to share a high sequence similarity, such predictions may increase the risk of occurrence of false positives [23, 24].

The primary goal of this study was to detect GPCR s in the predicted FG proteome, to select the best candidates for potential use to control this pathogen and to identify potential inhibitors. Several *in silico* predictive filters were used leading to the selection of one of the most relevant GPCR target. Prior to the rational screening of putative active compounds, the three-dimensional structure of this GPCR should be known. As no FG GPCRs’ 3D structures are presently available, we used homology modeling and molecular dynamics simulations in order to obtain a convincing model for the selected GPCR candidate. Then, stable conformations of this model were used to identify potential inhibitors using the virtual screening approach [25, 26].

## Results and discussion

### Identification of putative GPCRs

The 13,321 predicted protein sequences of *Fusarium graminearum* (Version 32) were submitted to the bespoke GPCRpipe, and only nine sequences were identified as putative GPCRs. After checking for the number of their transmembrane helices by TMHMM, HMMtop, and Phobius, only six proteins among the nine were found to contain the necessary 7 transmembrane helix (TM) feature confirmed by all three programs (Table 1). For the three others, as at least two programs predicted 7 TMs, we finally considered all the nine as putative GPCRs. Furthermore, all these predicted GPCRs presented an extracellular N-terminus and an intracellular C-terminus like other known GPCRs, strengthening, therefore, this selection. In the group of nine candidates, seven are in common with the previous annotation by Ma et al. (2010). Among them two (FGSG_02942 and FGSG_05404) are novel GPCR candidates coming from the stringent structural/function prediction (Table 1).

**Table 1:** Number of transmembrane helices determined by TMHMM, HMMtop and Phobius

Protein ID	# Helices		
	TMHMM	HMMtop	Phobius
FGSG_01861	7	7	7
FGSG_02655	6	7	7
FGSG_02942	7	7	5
FGSG_03023	7	7	7
FGSG_05006	5	7	7
FGSG_05239	7	7	7
FGSG_05404	7	7	7
FGSG_07270	7	7	7
FGSG_07716	7	7	7

### Functional classification and final selection

**Fig. 1:**
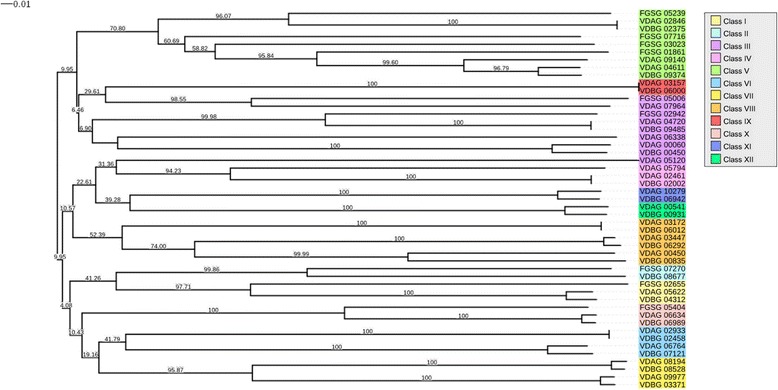
Phylogeny of nine putative GPCRs identified in *Fusarium graminearum* and 40 GPCRs identified by Zheng et al. in *Verticillium dahliae* and *Verticillium albo-atrum*. The unrooted tree with bootstrap value (10,000 repetitions) shown in every branch was constructed using the neighbor-joining method

As fungal GPCRs are associated with different functions, identification of these functions for the nine putative GPCRs was used as a first step to select the best target to inhibit fungal growth. Zheng et al. (2010) proposed a new classification of fungal GPCRs and classified 40 GPCRs from the ascomycete pathogens *Verticillium dahliae* and *Verticillium albo-atrum*, and we used these sequences to build the phylogenetic tree of the nine putative GPCRs (Fig. 1). FGSG_05239, FGSG_07716, FGSG_03023 and FGSG_01861 are present in the same branch as class V GPCRs. FGSG_05006 and FGSG_02942 are grouped into class III GPCRs. FGSG_02655 and FGSG_07270 are both pheromone receptors belonging respectively to class I and class II. The last GPCR, FGSG_05404, is similar to class X. To confirm this phylogeny relationship, a domain composition of each protein was determined using Pfam (Table 2) showing that all extracted domains are coherent with GPCRs functions: STE2 and STE3 are mating type pheromone receptor domains, Git3 is a glucose receptor, 7tm_1 and 7tm_2 are respectively rhodopsin and secretin receptors, dicty_CAR is a cyclic AMP receptor, and Lung_7-TM_R is a known seven transmembrane helix domain.

**Table 2:** Classes of the retained GPCRs identified in FG and Pfam domains

Protein ID	Conserved Pfam domain	Class [17]
FGSG_02655	STE2 (PF02116)	I. Ste2-like pheromone receptor
FGSG_07270	STE3 (PF02076)	II. Ste3-like pheromone receptor
FGSG_02942	Git3 (PF11710)	III. G protein-coupled glucose receptor regulating Gpa2
FGSG_05006		
FGSG_05239	7tm_1 (PF00001)	V. cAMP receptor like
FGSG_01861	7tm_2 (PF00002)	
FGSG_03023	Dicty_CAR (PF05462)	
FGSG_07716		
FGSG_05404	Lung_7-TM_R (PF06814)	X. PTM1-like GPCR

To identify potential new fungicide targets, an important step is to verify that the identified targets are not present in host organisms (principally wheat and corn) and humans. As shown in Table 3, for eight of the nine identified GPCRs, no similar protein was found in *Homo sapiens*, *Zea mays* or *Triticum* species. For the putative GPCR FGSG_05404 similar was found to a protein occurring in the three different tested species. As a consequence, FGSG_05404 was discarded from any further analyses.

**Table 3:** Similarity with other species

Protein ID	Blastp best results								
	*Zea mays*			*Homo sapiens*			*Triticum*.		
	E-value	Query-Cover	Ident	E-value	Query-Cover	Ident	E-value	Query-Cover	Ident
FGSG_02655	–	–	–	–	–	–	–	–	–
FGSG_07270	–	–	–	–	–	–	–	–	–
FGSG_02942	–	–	–	8e-03	46%	26%	–	–	–
FGSG_05006	–	–	–	5e-02	26%	25%	–	–	–
FGSG_05239	–	–	–	7e-03	41%	24%	–	–	–
FGSG_01861	4e-06	39%	26%	2e-04	41%	24%	–	–	–
FGSG_03023	–	–	–	2e-02	44%	24%	–	–	–
FGSG_07716	6e-05	85%	24%	3e-04	43%	25%	–	–	–
FGSG_05404	6e-33	70%	30%	9e-21	51%	28%	6e-29	73%	27%

Blastp best results: the nine identified GPCR vs *Homo sapiens*, *Zea mays & Triticum* (nr database). Only best results with an E-value lower than 1e-01 are displayed

From the eight remaining possible GPCR targets, only FGSG_02655, predicted to code for a class I pheromone receptor, was retained for entering into the molecular modeling pipeline. This protein contains a mating type pheromone receptor domain (PF02116). In fungi, one of the first steps in sexual reproduction is sexual pheromone reception by a mating type receptor. As sexual reproduction is a fundamental part of the FG disease cycle, FGSG_02655 is therefore considered a good choice for developing as a fungicide target [27]. Using a reverse genetics approach, FGSG_02655 has already been shown to be required for sexual mating and virulence in *F. graminearum.* Single gene deletion strains exhibit reduce female fertility and fewer mature perithecia were produced when strains were selfed [28, 29]. In addition, a separate study [30] confirmed the sexual mating defects and also showed the reduced ability of a delta FGSG_02655 strain to cause disease on wheat ears and maize cobs. Therefore, the selection of FGSG_02655 as a candidate fungicide target could be used to restrict *F. graminearum* growth and development at two distinct phases in its predominately monocyclic disease cycle.

### Homology modeling of FGSG_02655

**Fig. 2:**
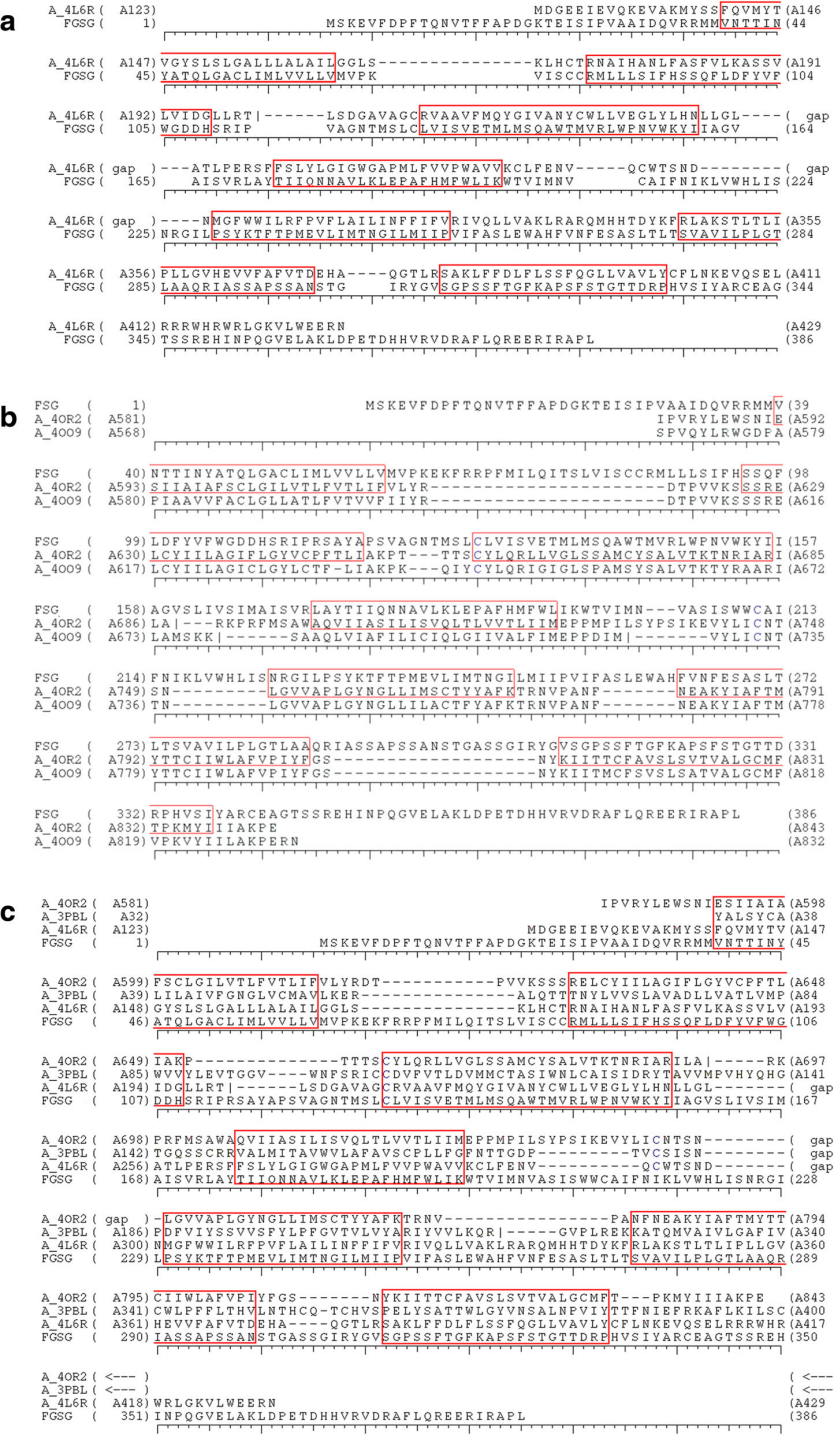
**a** Alignment of FGSG_02655 with a sequence of human glucagon G-protein coupled receptor (4L6R). This alignment was used to build the model 1. **b** Alignment of FGSG_02655 with sequences of human G protein-coupled metabotropic glutamate receptor 1 (4OR2) and human metabotropic glutamate receptor 5 (4OO9). This alignment was used to build the model. **c** Alignment of FSG_02655 with sequences of G protein-coupled metabotropic glutamate receptor 1 (4OR2), the human dopamine D3 receptor (3PBL) and human glucagon G-protein coupled receptor (4L6R). This alignment was used to build the model 3. Red squares correspond to transmembrane helix positions

The first step in homology modeling is to identify the most suitable templates to use to build the query 3D model. The sequence similarity between FGSG_02655 and known GPCRs’ PDB was found to be very low. Therefore, no structure could be used as a template. Instead the FGSG_02655 transmembrane helices were predicted using several tools (Table 4). Firstly, sequence similarity was not used to build the models as it is usually done in homology modeling. The 7 TM helices positions were used, aligning the TM sequences predicted for the FGSG_02655 query with those observed in the PDB templates. Moreover, we used supplementary information concerning the third transmembrane helix: in the structures used as templates as well as in the majority of PDB GPCR structures [31], a cysteine forms a disulfide bond with the second extracellular loop [12]. After analyzing these different transmembrane helix predictions, three models of FGSG_022655 were finally retained. The corresponding TM helices are highlighted in Table 4, and the corresponding sequence alignments are presented in Fig. 2a–c. As quality score determined by DOPE (discrete optimized protein energy) were similar (Table 5) for the 3 selected models, they were submitted to molecular dynamics (MD) simulations to check their stabilities.

**Table 4:** Transmembrane helix predictions for FGSG_02655

Prediction method	TM1	TM2	TM3	TM4	TM5	TM6	TM7
DAS	49–63	74–93	126–136	155–174	193–220	241–258	271–285
PRED-TMR	45–63	71–93	156–178	192–214	240–258	269–187	
HMMTOP	46–63	72–93	116–134	155–177	200–219	240–258	269–286
TMHMM	42–64	71–93	113–135	156–178	198–220	241–263	
GPCRHMM	41–63	72–93	114–135	152–171	192–214	232–258	263–283
PredictProtein	44–61	76–95	123–143	155–179	202–219	241–259	269–286
TOPCON	43–62	72–92	120–140	156–176	200–220	240–260	268–298
MINNOU	39–62	71–105	118–147	152–185	192–224	243–257	269–289
SOSUI	117–138	146–168	193–215	225–247	268–290	310–332	340–362
SPLIT	122–138	147–170	198–213	227–251	264–296	312–338	350–368
TMpred	127–146	155–175	198–216	237–256	277–296	322–338	350–368
Model 1	39–61	86–109	129–156	176–197	230–252	275–299	312–333
Model 2	39–61	95–118	129–156	173–195	225–247	263–287	311–337
Model 3	39–61	86–109	129–156	176–197	225–247	263–287	307–328

**Table 5:** DOPE score of the 3 selected models

Model	DOPE score
1	−34,627.5
2	−34,211.42
3	−34,766.89

Scores were calculated by the assess_dope function of Modeller

### Checking the stability of the models by molecular dynamics

The three protein models of FGSG_02655 selected from the previous step were subjected to a ten ns MD simulation to determine their stability as a GPCR. Evaluation of stability was realized by analyzing secondary structure evolution during the dynamics. The main focus of this analysis was the transmembrane helices: if the seven transmembrane helices were not broken during the Molecular Dynamics, then we consider the model as stable. The analysis of secondary structure evolution shows that for two protein models (model 1 and 2), transmembrane helices were not retained during the simulation. In model 2, the third transmembrane helix broke during the equilibration and after 4 ns TM helix 7 was broken. Both helices did not re-form during the MD. Concerning model 1, helix 4 was broken at the beginning of the Molecular Dynamics and did not reform during the simulation. These observations indicate that model 1 and model 2 were not stable and were not considered further.

**Fig. 3:**
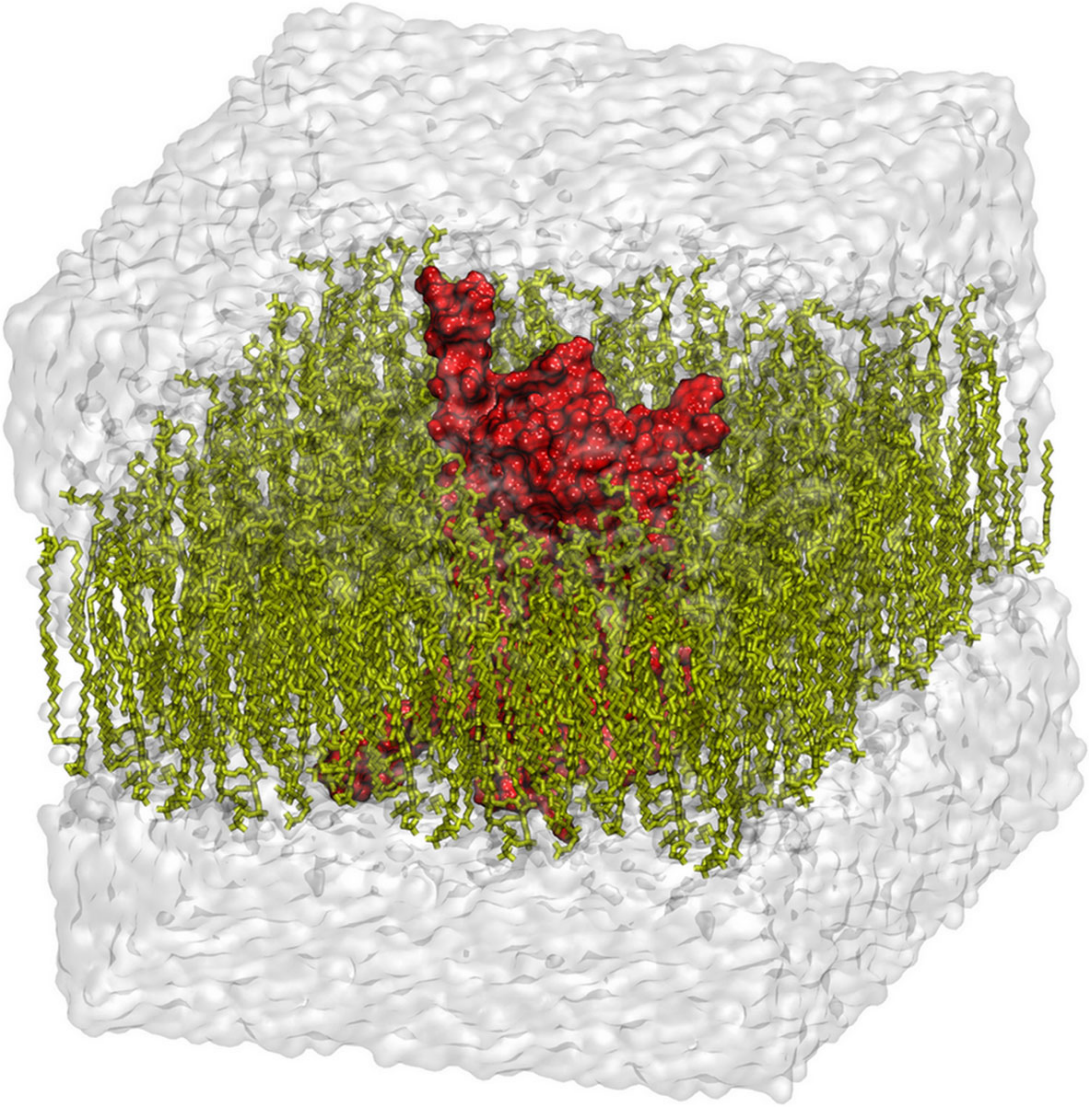
The appearance of the initial Model 3. The red color indicates the GPCR, the olive green color indicates the membrane lipids and the grey color indicates the water box

For model 3 (Fig. 3), all the secondary structures were stable during the original simulation. As this model appeared to be stable, we prolonged the MD simulation until 100 ns.

### Choice of the final model

**Fig. 4:**
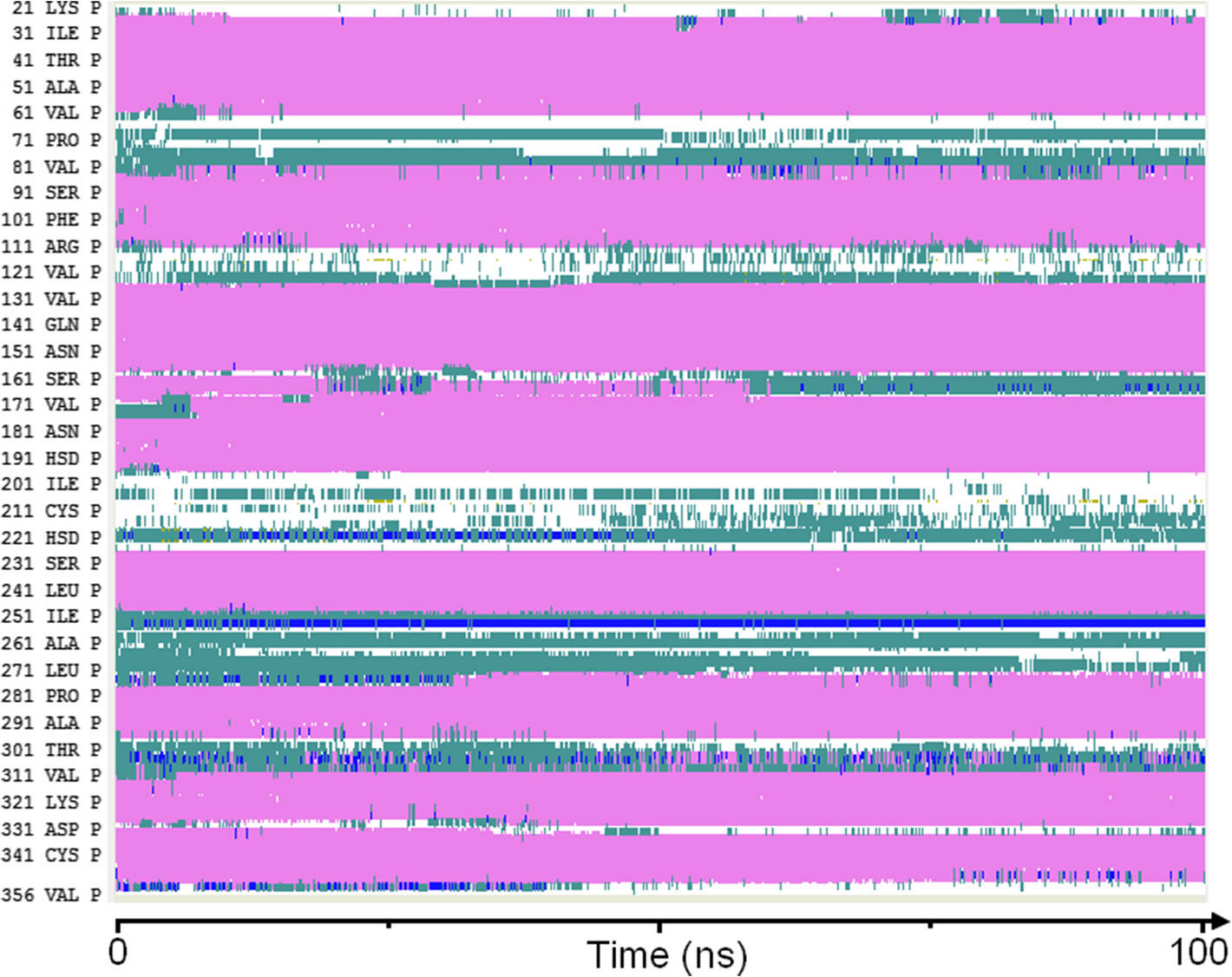
Evolution of model 3 secondary structure during the 100 ns of Molecular Dynamics simulation. Turns are represented in green, α helices in pink, 3–10 helices in blue and coils in white

**Fig. 5:**
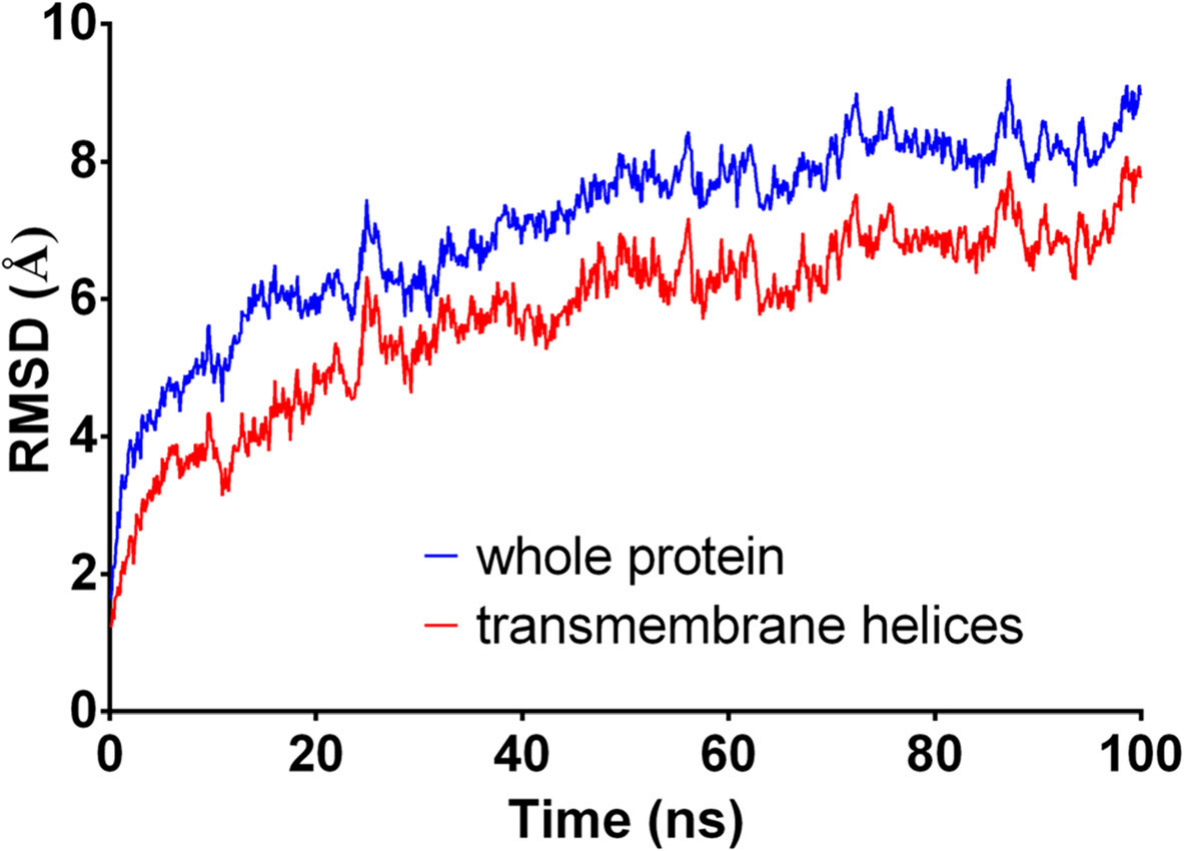
Root mean square deviation (RMSD) values for the whole FGSG_02655 protein compared with RMSD values from the seven transmembrane helices

**Fig. 6:**
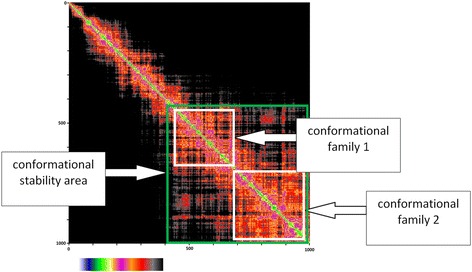
Root mean square deviation (RMSD) map showing the conformational behavior of the protein during the 100 ns MD. The color scale is given below, the black color corresponding to dissimilar conformations (RMSD > 3.5 Å) and white corresponds to identical conformations (RMSD = 0 Å). The two conformational families 1 and 2 found as stable structural states are highlighted by the white squares

**Fig. 7:**
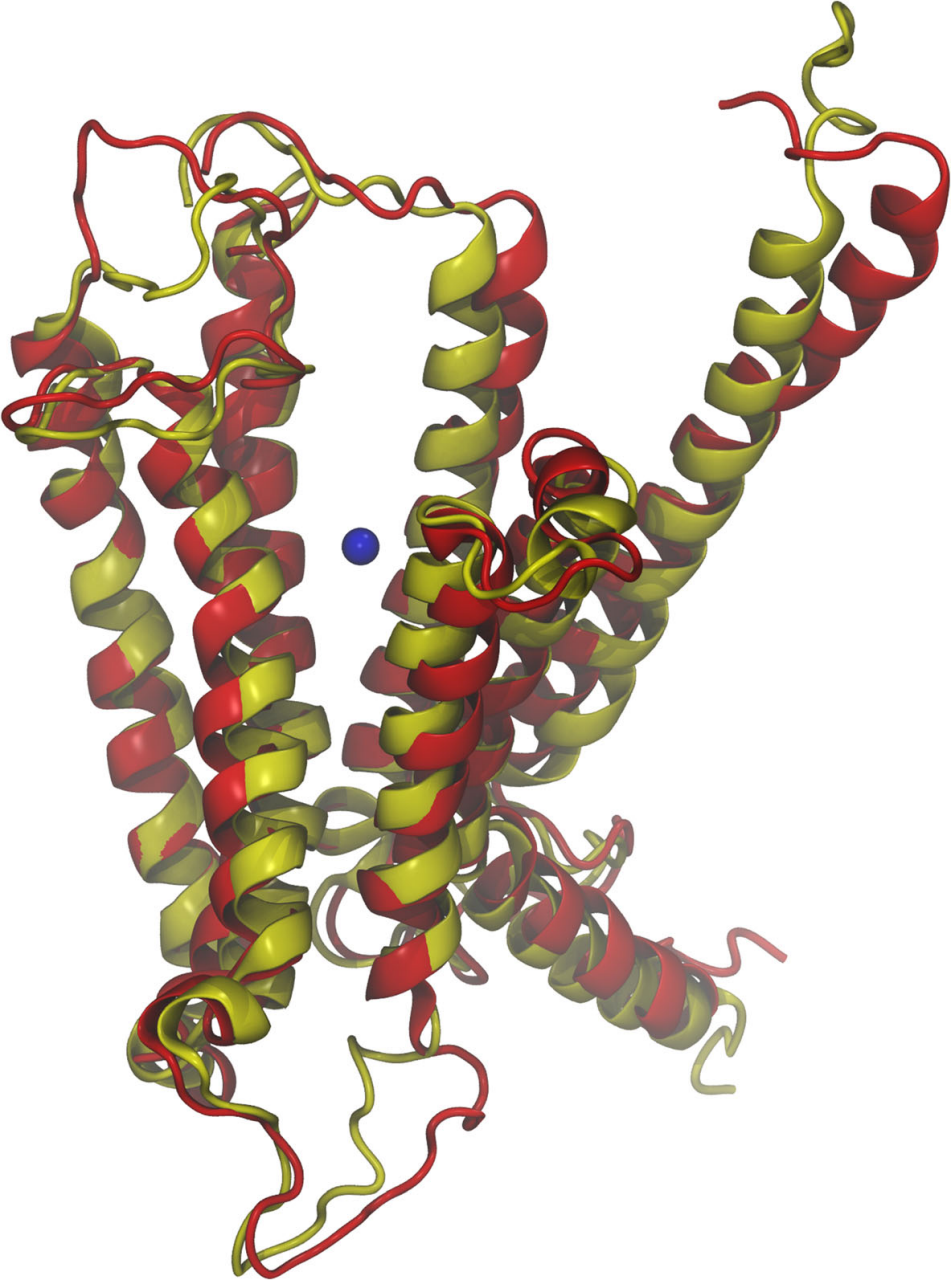
Structural alignment of the conformational families. The first conformation is in yellow and the second in red. The center of the binding pocket is in blue

The timeline analysis of the secondary structures during the 100 ns of MD simulation showed that the third model is stable during the whole trajectory and especially the transmembrane helices (Fig. 4). Then, each frame of the trajectory was aligned to the first one based on the protein backbone. Root-mean-square deviation (RMSD) analysis was performed for both the whole protein and its seven transmembrane helices (Fig. 5). It appears from these simulations that a very stable conformational regime was obtained after 42 ns, mostly due to a rearrangement of the TM helices within the palmitoyloleoylphosphatidylcholine (POPC) bilayer. During the remaining 58 ns, the protein conformation fluctuates between two quite similar conformational families (Fig. 6). Concerning these two conformations, three of their transmembrane helices had the same positions (helices three, five and seven), two were shorter in the first conformation (helices one and six) and two others were shorter in the second conformation (helices two and four) (Fig. 7). For the first family, the distance between the α carbon of the N-terminal residue and the nearest POPC atom was around 28 Å and 20 Å for the C-terminal side. For the second conformation, these same distances were both 13 Å, showing that the first conformation family was the outermost of the membrane. The representative 3D structures of each of these families can be used for performing a structure-based virtual chemical screen using the ensemble docking procedure.

### Virtual screening

**Fig. 8:**
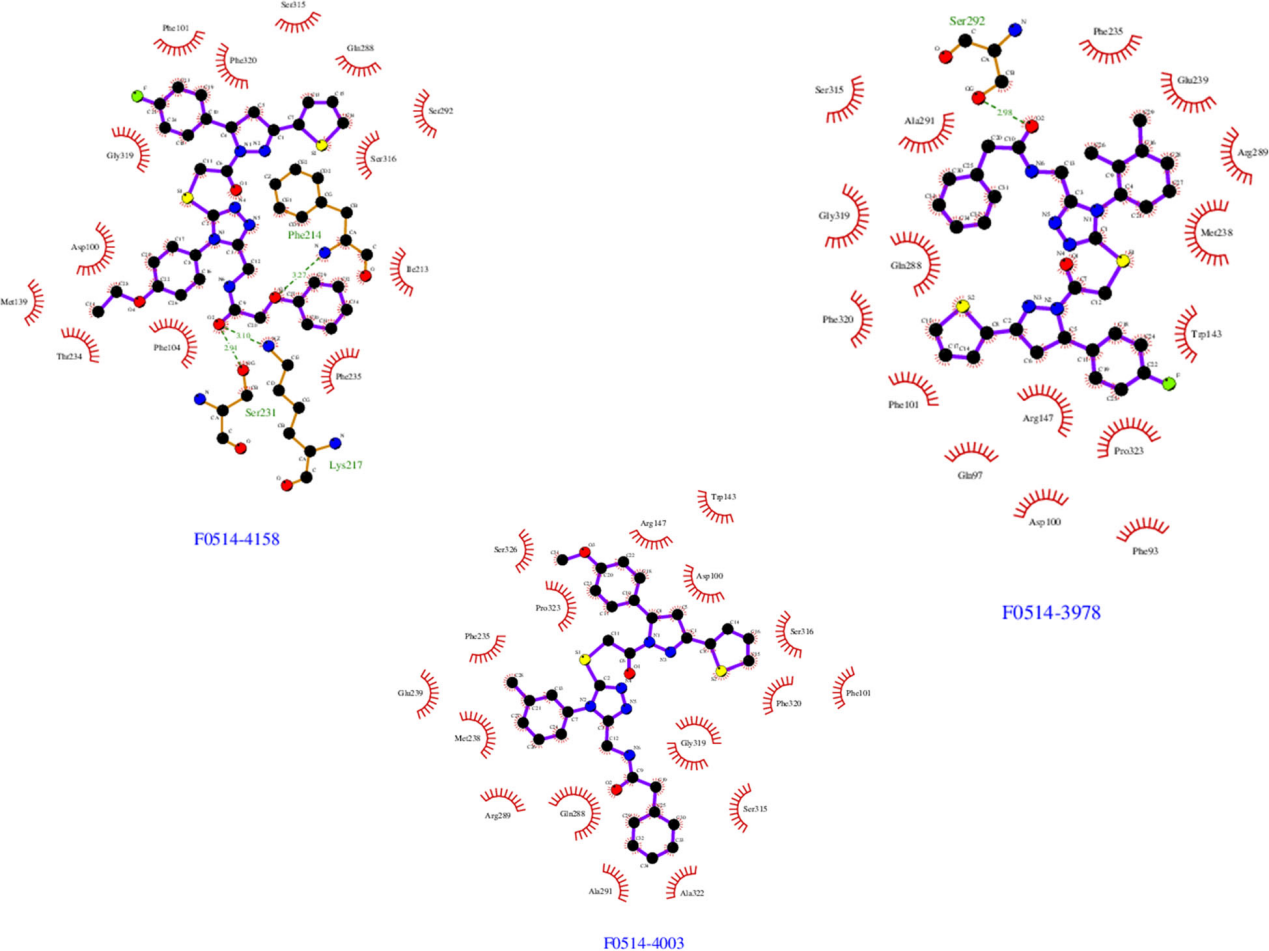
Interactions found between the protein and the ligands for the 3 highest GOLD score retained compounds

From the ensemble docking campaign using these two main conformers, we retained only the top 30 compounds from the complete GOLD score list to further analysis. After removing possible toxic molecules and compounds outside the pocket, we retained only 10 molecules (Table 6) for possible biological testing. It should be noted that the majority of the compounds bound the first conformation. The chemical formulas and names of these molecules are shown on Additional file 1: Table S1. The positioning of the 3 best-score retained compounds within the binding site is presented on Fig. 8. Looking at the protein/ligand interaction found, it appears that each ligand had specific interactions with the protein: for example, compound F0514-4158 interacts with Phe_214_ Lys_217_ and Ser_231_, while the molecule F0514-3978 interacts only with Ser_292_.

**Table 6:** GOLD scores for the finally selected compounds (in bold)

Rank	Score (PLP)	ID dataset	#Atoms outside	Frame	Toxicity
*1*	*110.31*	*F1044-0055*	*25*	*525*	
*2*	*109.35*	*F1044-0055*	*18*	*840*	
3	107.46	**F0514-4158**	10	525	
4	106.61	**F0514-3978**	4	525	
*5*	*106.59*	*F0514-5375*	*18*	*525*	
6	105.88	**F0514-4003**	8	525	
7	105.7	**F0617-0172**	16	840	
8	105.67	F0520-1906	0	840	+
9	105.66	**F3407-3991**	0	840	
10	105.57	**F0514-4846**	12	525	
11	105.5	**F0514-0510**	10	525	
12	105.35	**F0514-3894**	5	525	
13	105.27	**F0514-5342**	13	525	
14	105.24	**F0514-4074**	0	840	
15	105	F0514-3894	8	525	

Molecules in italics are not selected because of their high number of atoms outside the pocket

## Conclusion

For this study we have used a bespoke analysis pipeline called GPCRpipe followed by a stepwise funnel strategy to identify, select and model one putative GPCR protein that could be of possible use as drug target to design new compounds active against *Fusarium graminearum*. This iterative search procedure is innovative because it has combined the use of genomics and molecular protein modeling approaches. Considering the 117 GPCRs candidates previously predicted in the FG genome sequencing [22] and nine sequences initially obtained by our strategy, we anticipated that a few false positives were retained. Therefore, we used additional filters based on structural and functional criteria to predict the most authentic GPCR candidates. Our selection of the nine resulting FG GPCR candidates was based not only on sequence similarity but on Molecular Dynamic modelling of the 3D protein structure. In addition, literature mining and a phylogeny analysis was used to explore the potential biological processes associated/proposed for each GPCR. For the final sequence retained, namely FGSG_02655, the molecular dynamics simulations proved to be an efficient method to choose between several alignments between the putative FG GPCR query and the template used in the homology modeling process as only one of the three predicted models came through the extended 100 ns MD stimulation intact. Furthermore, a method developed to model GPCR was recently published [32] and may be interesting to use in future studies.

The receptor conformational flexibility highlighted by the MD simulation on the retained final robust model was used for running next an efficient ensemble docking structure-based virtual screening which provides interesting hypothetical hits to be now proposed for experiments. The whole *in silico* selection funnel presented in this study provide an example of an integrated process merging genomics, structural bioinformatics and drug design and leading to propose valuable and innovative solutions to a world-wide threat to grain producers and consumers.

## Methods

**Fig. 9:**
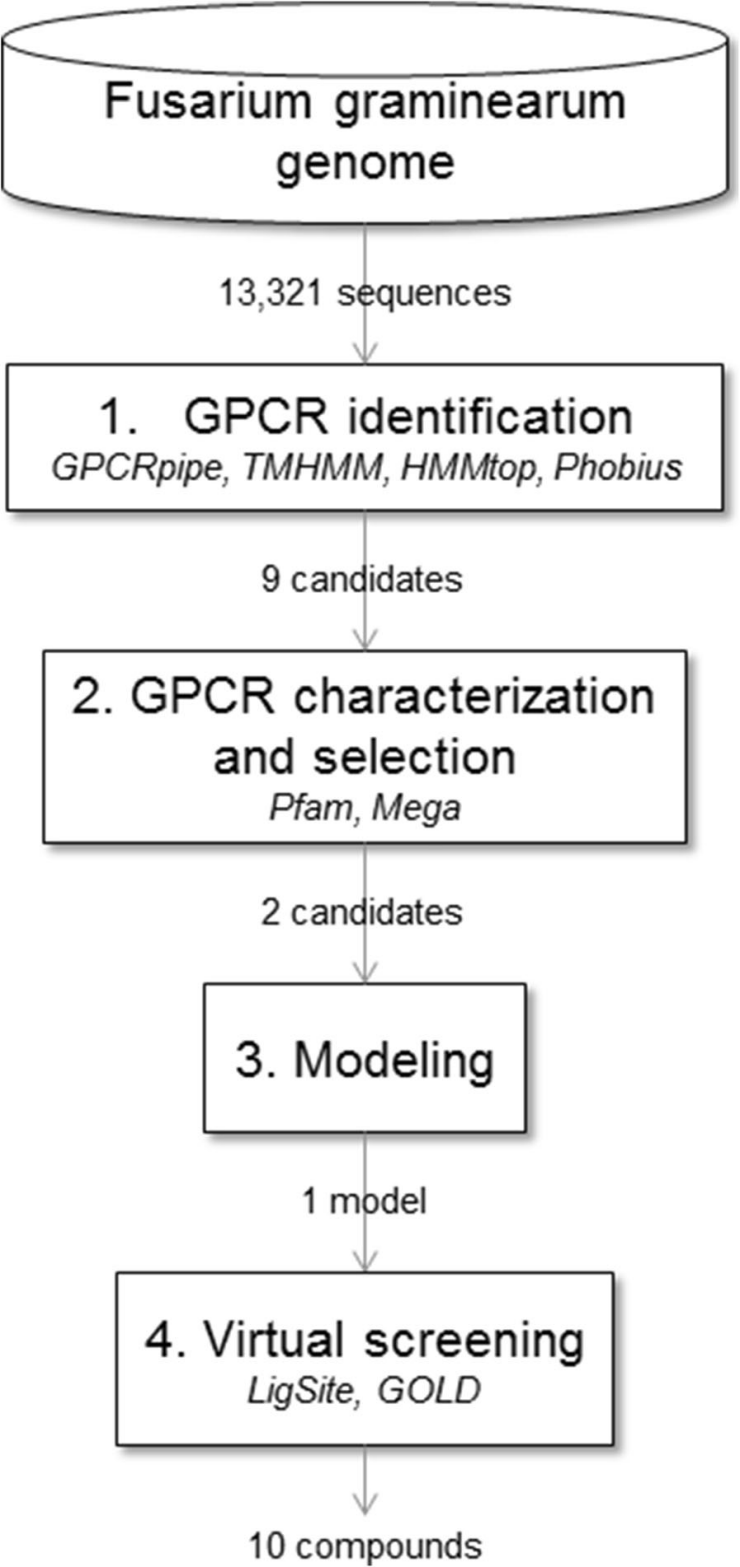
Proposed strategy to predict and identify novel GPCR through bioinformatics approach. Programs used for each step are in italic

Our in silico experimental approach was composed of three main steps as illustrated in Fig. 9. The first step was related to the identification of GPCRs in the FG genome using several GPCRs prediction tools. Next, having identified putative GPCRs, these were classified according to their function to select a limited set of possible targets for designing new and innovative compounds against FG. The third step of this funnel consisted of a molecular modeling approach to building the three-dimensional models of these targets. The last step corresponds the virtual screening. Binding pockets were detected in stable models identified in the previous step. Then, a large compound library was used with a docking program to find putative inhibitors. All calculations were performed on a 64 cores computer.

- Step 1: GPCR identification in FG proteome.
  The *Fusarium graminearum* genome was firstly published in 2007 [33]. The complete proteome of *Fusarium graminearum* PH-1 assembly FG3 (13,321 proteins) was downloaded from the BROAD Institute *Fusarium* comparative database [34].
  FG putative GPCRs were firstly detected using the GPCRpipe program [24]. Two methods in GPCRpipe were used for the detection of GPCR. Based on Hidden Markov Model, the first step was designed for the detection of GPCR. The second one is a library that consists of 39 Pfam profile HMMs (specific to different families of GPCR). We used the GPCRpipe “AND” method, meaning that a GPCR was predicted only if the two methods confirmed the prediction. This choice resulted in a reduced number of GPCRs predictions and limited the number of false positive predictions.
  The next step, to validate the GPCRpipe predictions, involved using three transmembrane prediction softwares namely HMMtop [35], TMHMM [36] and Phobius [37]. The first two are the best-known transmembrane prediction methods, and Phobius was reported to perform comparably [37, 38].
- Step 2: GPCR characterization, selection, and annotation.
  The functional classification of the GPCRs identified in *Fusarium graminearum* was realized using a PfamA analysis [39]. In parallel, the phylogenetic tree of these GPCRs and the 40 identified GPCRs in *Verticillium dahliae* and *Verticillium albo-atrum* [17] was built. This tree was built by Mega 6.0 using the Neighbor-Joining method and 10,000 repetitions.
- Step 3: 3D Model Building.
  - Homology modeling
    The construction and validation of the various homology models of GPCRs is still a challenge [40] and requires not only the sequence alignments but would also include structure-based alignments. This approach has been proved successful in many studies [41, 42]. Nowadays, the structure of 20 different class A, two class B, two class C, and one frizzled GPCRs are available in the PDB [43], providing a reasonable set of possible templates to be used.
    The choice of the proper template is crucial for ensuring the validity of the homology model. For that, several strategies are proposed, and many questions still remain [44]: for example, do we have to choose a single template, and in this case how to select it, or a set of templates? Such a decision can be difficult as contradictory results were obtained such as the ones claiming that a single well-chosen template is better than a set [45] versus the ones with opposite conclusions [46]. Moreover, it appears clearly from recent studies that the accuracy of the model greatly depends on the phylogenetic tree proximity of the template and the target [47].
    Consequently, when considering (i) the remarks above, (ii) the conservation of the 7TM bundles in all GPCRs and the observed deformation of its helices [48], and (iii) the sequence conservation of several motifs [49] we decided to start our homology modeling phase using both phylogenetic data (for selecting the most suitable template) and helix predictions information (to align the TM helices sequences between the template and the target). Next the loops connecting the TM helices were added to the models obtained this way, considering also the constraint of the possible disulfide bridges [50, 51].
    To build our models, transmembrane helix positions were determined by 11 transmembrane helix predictors (Additional file 2: Table S2 [23, 35, 52–59]). Then, our models were constructed by aligning transmembrane helices instead of similar amino acids. Moreover, crystal structures of several GPCRs, as well as experimental evidence, have shown the presence of a disulfide bond linking transmembrane helix 3 (TM3) to the second extracellular loop (ECL2) [60] and we used this additional information positioning this cysteine residue at the top of TM3. Finally, the position of the conserved motifs was also used for our selection process [61]. The homology modeling task was performed using the MODELLER program with its default settings [62]. Additionally, the automatic loop refinement method available in MODELLER was used. The DOPE score from MODELLER was used to estimate model quality.
  - Molecular dynamics (MD)
    The next steps using Molecular Dynamics simulations were required to refine the preliminary crude models and then analysis the stability of the GPCR within the membrane [63]. MD is now commonly used to validate homology models, especially in the GPCRs field [64–67].
    For this purpose, we used molecular dynamics simulation on the receptor homology models that were embedded in a fully hydrated POPC bilayer [68]. No ligand was positioned within the receptor at this level as it has been shown [47] that the presence of a ligand does not change the accuracy of the structure produced. Initially, the receptors models were positioned across the equilibrated bilayer while seeking to match the hydrophobic protein segments with the layer formed by the lipid hydrocarbon tails. Lipids overlapping with the protein complex were deleted, leaving a bilayer consisting of 357 POPC molecules. To ascertain that the cytoplasmic and extracellular loops did not interact, an amount of 35,479 water molecules was added, as well as 10 counterions to make the whole system-neutral, thus making a total number of atoms equal to 159,461. The complete system, represented in Fig. 3 was replicated periodically in the three directions of space, with a repeat distance of ∼ 120 Å.
    The MD simulations were carried out in the isobaric-isothermal ensemble, maintaining the pressure and the temperature of 1.0 atm and 300.0 K, respectively, using Langevin dynamics and the Langevin piston approach. The MD program NAMD [63] was employed in conjunction with the CHARMM27 force field [69] to describe the receptor, the lipid bilayer, and the water molecules. Coulomb forces were evaluated with the particle-mesh Ewald method. The equations of motion were integrated with a 1-fs time step, using the r-RESPA algorithm to update short- and long-range contributions at different frequencies.
    Each system was energy minimized and then equilibrated (3 ns) before recording trajectories. All MD trajectory frames were recorded at 1 ps intervals, for a total of 10 ns simulation. Model stabilities were then checked by analyzing secondary structure evolution during the MD simulation. If at least one transmembrane helix broke, then the model was not considered stable. For the stable models obtained, the simulations were extended to reach a 100 ns simulation time.
  - MD simulation analysis
    Once the MD simulation was completed, all the frames were aligned by only take into account the protein backbone. RMSDs were calculated by the “RMSD trajectory tool” plugin from Visual Molecular Dynamics (VMD) [70]. RMSD maps were built using a previously developed in-house Tool Command Language (TCL) script.
- Step 4: Virtual screening
  - Ligand library
    The chemical libraries used for the virtual screening were the GPCR Targeted Libraries (11,571 compounds) from Life chemicals. These libraries contained compounds for sixteen types of GPCRs.
  - Pocket detection and analysis
    For each stable conformer detected thanks to the MD simulation, the coordinates of the binding pocket center were identified using LigSite [71].
  - Ensemble docking
    For the docking, we used the docking program GOLD [72] which has been considered as one of the best docking software [73]. Because several stable conformations were identified, we used the ensemble docking possibility available in GOLD. The use of such conformational ensembles was considered as an improved strategy in structure-based docking calculations [74]. For each docking, 100 starting ligand conformers were used in GOLD. All target conformers used were aligned in a common reference system and the center of the pocket cavity is an average of the individual centers found in each conformation. A sphere of 15 Å was selected to define the binding region around this center.
  - Toxicity
    In order to remove probable toxic molecules, the side effects of the finally identified compounds were detected using toxicity predictors such as PAINS-remover, Badapple and Protox webservers [75–77].

## Additional files

Additional file 1: Table S1. Name and 2D structure of the 10 retained compounds (PDF 108 kb)
Additional file 2: Table S2. Programs used for detecting transmembrane helix positions (PDF 34 kb)

## Abbreviations

3D: Tri-dimensional
DOPE: Discrete optimized protein energy
FG: *Fusarium graminearum*
FHB: *Fusarium* head blight
GPCR: G-protein coupled receptor
MD: Molecular dynamics
PDB: Protein databank
RMSD: Root mean square deviation
TCL: Tool command language
TM: Transmembrane
VMD: Visual molecular dynamics
