# Supplementary material for: GPCRs from fusarium graminearum detection, modeling and virtual screening - the search for new routes to control head blight disease
Source: BMC Bioinformatics. 2016 Dec 15;17(Suppl 18):463. doi: 10.1186/s12859-016-1342-9 (PMC5249037; doi:10.1186/s12859-016-1342-9)
Supplement: Additional file 1: Table S1. — Name and 2D structure of the 10 retained compounds (PDF 108 kb) [file 12859_2016_1342_MOESM1_ESM.pdf]

Additional table 1. Name and 2D structure of the 10 retained compounds.

Rank	ID Life Chemicals	Name	2D structure
1	F0514-4158	N-{[4-(4-ethoxyphenyl)-5-({2-[(5S)-5-(4-fluorophenyl)-3-(thiophen-2-yl)-4,5-dihydropyrazol-1-yl]-2-oxoethyl}sulfanyl)-1,2,4-triazol-3-yl]methyl}-2-phenoxyacetamide	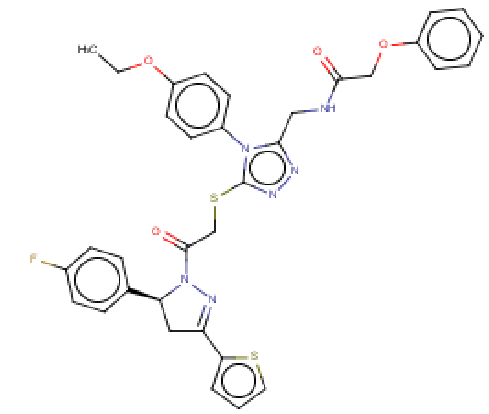
2	F0514-3978	N-{[4-(2,3-dimethylphenyl)-5-({2-[(5S)-5-(4-fluorophenyl)-3-(thiophen-2-yl)-4,5-dihydropyrazol-1-yl]-2-oxoethyl}sulfanyl)-1,2,4-triazol-3-yl]methyl}-2-phenylacetamide	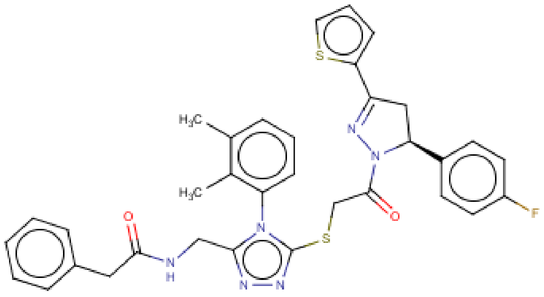
3	F0514-4003	N-{[5-({2-[(5S)-5-(4-methoxyphenyl)-3-(thiophen-2-yl)-4,5-dihydropyrazol-1-yl]-2-oxoethyl}sulfanyl)-4-(3-methylphenyl)-1,2,4-triazol-3-yl]methyl}-2-phenylacetamide	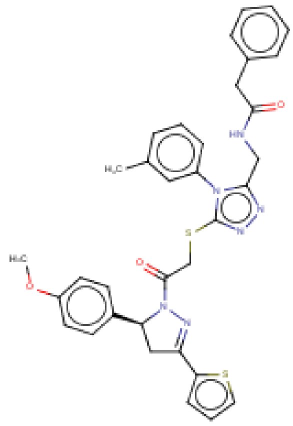
4	F0617-0172	N-{[5-({2-[(5S)-5-(4-methylphenyl)-3-(thiophen-2-yl)-4,5-dihydropyrazol-1-yl]-2-oxoethyl}sulfanyl)-4-(2-phenylethyl)-1,2,4-triazol-3-yl]methyl}thiophene-2-carboxamide	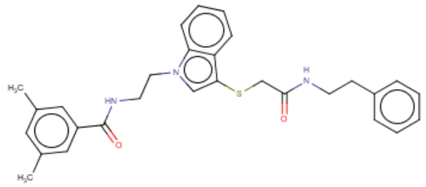

5	F3407-3991	N-{[4-methyl-5-({2-[(5S)-5-(4-methylphenyl)-3-(thiophen-2-yl)-4,5-dihydropyrazol-1-yl]-2-oxoethyl}sulfanyl)-1,2,4-triazol-3-yl]methyl}-2-phenoxyacetamide	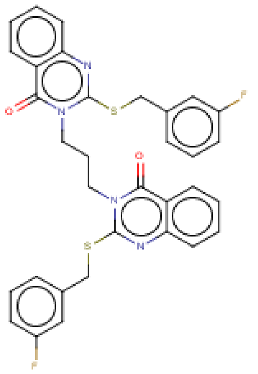
6	F0514-4846	N-{[4-benzyl-5-({2-[(5S)-5-(4-methylphenyl)-3-(thiophen-2-yl)-4,5-dihydropyrazol-1-yl]-2-oxoethyl}sulfanyl)-1,2,4-triazol-3-yl]methyl}-2-phenylacetamide	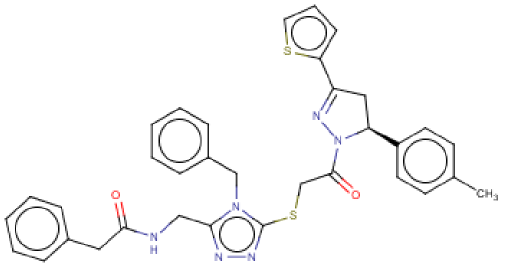
7	F0514-0510	3-fluoro-N-{[5-({2-[(5S)-5-(4-fluorophenyl)-3-(thiophen-2-yl)-4,5-dihydropyrazol-1-yl]-2-oxoethyl}sulfanyl)-4-(4-nitrophenyl)-1,2,4-triazol-3-yl]methyl}benzamide	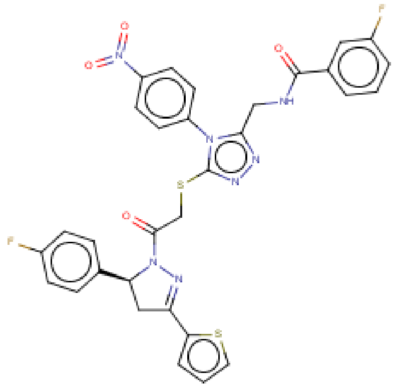
8	F0514-3894	N-{[5-({2-[(5S)-5-(4-fluorophenyl)-3-(thiophen-2-yl)-4,5-dihydropyrazol-1-yl]-2-oxoethyl}sulfanyl)-4-phenyl-1,2,4-triazol-3-yl]methyl}-2-phenylacetamide	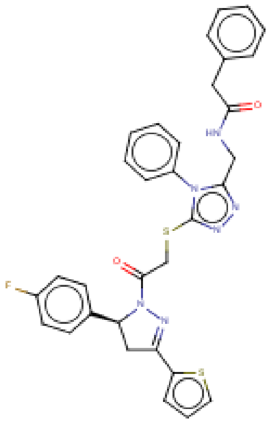

9	F0514-5342	N-{[5-({2-[(5S)-5-(4-fluorophenyl)-3-(thiophen-2-yl)-4,5-dihydropyrazol-1-yl]-2-oxoethyl}sulfanyl)-4-(2-phenylethyl)-1,2,4-triazol-3-yl]methyl}-2-phenoxyacetamide	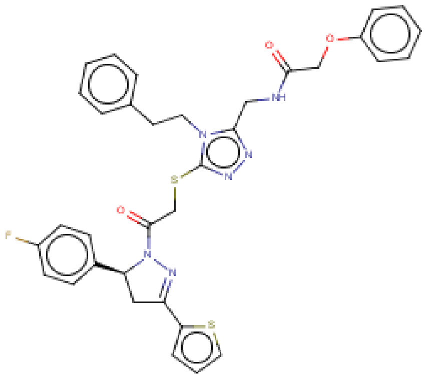
10	F0514-4074	N-{[4-(4-fluorophenyl)-5-({2-[(5S)-5-(4-fluorophenyl)-3-(thiophen-2-yl)-4,5-dihydropyrazol-1-yl]-2-oxoethyl}sulfanyl)-1,2,4-triazol-3-yl]methyl}-2-phenoxyacetamide	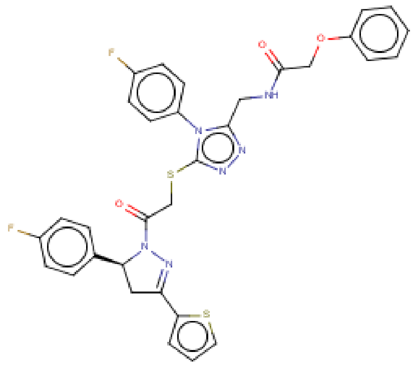
