# Supplementary material for: GPCRs from fusarium graminearum detection, modeling and virtual screening - the search for new routes to control head blight disease
Source: BMC Bioinformatics. 2016 Dec 15;17(Suppl 18):463. doi: 10.1186/s12859-016-1342-9 (PMC5249037; doi:10.1186/s12859-016-1342-9)
Supplement: Additional file 2: Table S2. — Programs used for detecting transmembrane helix positions (PDF 34 kb) [file 12859_2016_1342_MOESM2_ESM.pdf]

Additional Table 2. Programs used for detecting transmembrane helix positions.

Program	URL
**DAS**	www.sbc.su.se/~miklos/DAS
**GPCRHMM**	http://gpcrhmm.sbc.su.se/tm.html
**HMMTOP**	www.enzim.hu/hmmtop
**MINNOU**	http://minnou.cchmc.org
**PredictProtein**	www.predictprotein.org
**PRED-TMR**	http://athina.biol.uoa.gr/PRED-TMR
**SOSUI**	http://harrier.nagahama-i-bio.ac.jp/sosui
**SPLIT**	http://split4.pmfst.hr/split/4
**TMHMM**	www.cbs.dtu.dk/services/TMHMM
**TMpred**	www.ch.embnet.org/software/TMPRED_form.html
**TOPCONS**	http://topcons.cbr.su.se
